# Genetics of Cardiovascular Disease: How Far Are We from Personalized CVD Risk Prediction and Management?

**DOI:** 10.3390/ijms22084182

**Published:** 2021-04-17

**Authors:** Michal Vrablik, Dana Dlouha, Veronika Todorovova, Denes Stefler, Jaroslav A. Hubacek

**Affiliations:** 1 3rd Department of Internal Medicine, General University Hospital and 1st Faculty of Medicine, Charles University, 11636 Prague, Czech Republic; Veronika.Todorovova@vfn.cz (V.T.); jahb@ikem.cz (J.A.H.); 2 Experimental Medicine Centre, Institute for Clinical and Experimental Medicine, 14021 Prague, Czech Republic; dadl@ikem.cz; 3 Department of Epidemiology and Public Health, Institute of Epidemiology and Health Care, University College London, London WC1E 7HB, UK; denes.stefler@ucl.ac.uk

**Keywords:** cardiovascular disease, gene, interaction, polymorphism, gene score, epigenetic

## Abstract

Despite the rapid progress in diagnosis and treatment of cardiovascular disease (CVD), this disease remains a major cause of mortality and morbidity. Recent progress over the last two decades in the field of molecular genetics, especially with new tools such as genome-wide association studies, has helped to identify new genes and their variants, which can be used for calculations of risk, prediction of treatment efficacy, or detection of subjects prone to drug side effects. Although the use of genetic risk scores further improves CVD prediction, the significance is not unambiguous, and some subjects at risk remain undetected. Further research directions should focus on the “second level” of genetic information, namely, regulatory molecules (miRNAs) and epigenetic changes, predominantly DNA methylation and gene-environment interactions.

## 1. Introduction

**Figure 1:**
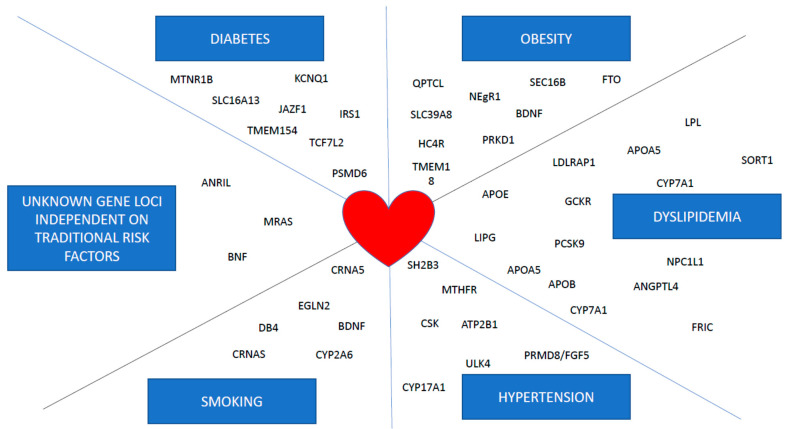
Genetic predisposition CVD. There are five nongenetic risk factors of CVD and all are under significant genetic control. Some selected genes with SNPs influencing mentioned risk factors and subsequently CVD are shown. Probably highest number of SNPs is associated with dyslipidemia, most powerful variants are associated with diabetes and obesity and the smallest effects have been observed in field of hypertension. Interestingly, smoking is by far the strongest predictor of CVD, but the list of genes associated with smoking behaviour is short and there is a lack of studies focused on these genes in CVD patients.

Cardiovascular disease (CVD) remains the most common cause of death in the majority of countries worldwide [1]. It includes coronary heart disease, cerebrovascular disease, peripheral arterial disease, rheumatic and congenital heart diseases, and venous thromboembolism (www.who.int/cardiovascular-disease#tab=tab_1/ accessed on 5 March 2021). Death rates from coronary heart disease (CHD) and stroke have generally been higher in Central and Eastern Europe (CEE) [2]. A decline in CVD mortality has been reported in most European countries; however, the decline in CVD mortality in CEE countries started substantially later [3]. Molecular genetics and pharmacogenetics play a key role in the diagnosis, prevention, and treatment of CVD. Genetic testing is used to identify the underlying genetic etiology in patients with suspected cardiovascular disease such as hypertrophic cardiomyopathy or familial hypercholesterolemia, and to determine who in the family has inherited the causal variant and is therefore at risk of developing CVD. Genetic testing should be carried out in well-phenotyped individuals and coupled with comprehensive family evaluation to aid in interpretation and application of the results [4]. Molecular genetics technologies applied to cardiovascular studies have enabled chromosome mapping and the identification of many genes involved in primary etiology (Figure 1).

Coronary artery disease and other common, complex cardiovascular diseases are heritable to variable extents. Our understanding of how DNA variants connect to function and how genetics may translate to the clinic has increased significantly over the past two decades [5]. In the following review, we present important examples of the genetic modification of CVD risk as well as the gene-environment and gene-pharmacotherapy relationships with regard to future use of these findings in personalized medicine.

## 2. Monogenic vs. Polygenic Determination

The majority of CVDs and CVD risk factors have polygenic backgrounds; thus, the interplay between environmental and lifestyle risk factors with risk alleles of dozens of polymorphisms is involved. However, monogenetic conditions can lead to severe premature CVD and early death if unrecognized and untreated.

The most common monogenic disease leading to premature CVD is familial hypercholesterolemia (FH). FH has a frequency of approximately 1:200 and is caused primarily by mutations within the LDL receptor (*LDLR*), apolipoprotein B (*APOB*), and *PCSK-9* genes [6]. The relative frequencies of monogenic variants might vary slightly among different populations, but the most frequent are mutations in *LDLR* [7,8]. Although more than 2900 *LDLR* mutations have been identified, approximately one-thousand mutations are considered to be the cause of FH. In contrast with single missense mutations in the *APOB* gene, pathogenic and likely pathogenic mutations in the *LDLR* gene are mostly exonic substitutions and missense rearrangements [9,10].

Hypertrophic cardiomyopathy (HCM) is the most common familial heart disease with vast genetic heterogeneity. Mutations in 11 or more genes encoding proteins of the cardiac sarcomere (>1400 variants) are responsible for (or associated with) HCM. Genetic testing also allows expansion of the broad HCM disease spectrum and diagnosis of HCM phenocopies with different natural history and treatment options, but is not a reliable strategy for predicting prognosis [11].

Other monogenic CVDs are rather rare in comparison to FH, such as sitosterolemia or Marfan syndromes, which occur with frequencies of less than 1:1000, and the frequencies of many other diseases are so rare that they have not even been determined (Table 1) (for a detailed review, see [11]).

**Table 1:** Selected examples of monogenic cardiovascular diseases.

Gene(s)	CVD	Manifestation	Frequency
*LDLR, APOB, PCSK9*	Familial hypercholesterolemia	High concentrations of LDL and total cholesterol; xanthomas; arcus lipoides cornae; xanthalesmas; coronary heart disease	1:200–250
*ABCG5, ABCG8*	Sitosterolemia	High plasma sitosterol, campesterol; hypercholesterolemia; premature coronary heart disease; xanthomas	1:2000
*MYH7, MYBPC3, TNNT2, TPM1, MYL2, MYL3, PLN,*	Hypertrophic cardiomyopathy	Hypertrophy of left ventricle, shortness of breath, diastolic dysfunction, left ventricular outflow ischemia	1:500
*PKP2, DSP, DSG2, JUP, TMEM43*	Arrhythmogenic right ventricular cardiomyopathy	Ventricular arrhythmias, right ventricular cardiomyopathy	1:5000
*MYH7, MYBPC3, TNNT2, MYH6, MYPN, ANKRD1, RAF1, DES, DMD*	Familial dilated cardiomyopathy	Diastolic dysfunction, left ventricular hypertrophy, atrial fibrillation, congestive heart failure	1:2500
*FBN1, TGFBR1, TGFBR2, SMAD3, TGFB2, TGFB3, SKI*	Marfan’s syndrome	Aortic aneurysm or dissection, valvular heart disease, enlargement of the proximal pulmonary artery, congestive heart failure, arrhythmias	1:5000
*ACTA2, FBN1, MYH11, TGFBR1/2, LOX, COL3A1, TGFB2/3*	Thoracic aortic aneurysm and dissection	Chest pain, renal cysts, thumb-palm sign, temporal arteritis, bicuspid aortic valve, abdominal aneurysm, intracranial aneurysm,	unknown
*BMPR2, BMPR1B, CAV1, KCNK3, SMAD9, ACVRL1, ENG, EIF2AK4*	Pulmonary arterial hypertension	Right ventricular failure, impaired brachial artery flow-mediated dilation, increased pulmonary vascular resistance	15:1,000,000
*KCNQ1/H2/E1/J2, SCN5A, CAV3, CALM1/2*	Long QT syndrome	Malignant arrhythmia, palpitations, syncope, anoxic seizures secondary to ventricular arrhythmia	1:2000
*KCNH2*	Short QT syndrome	Abbreviated QTc interval on the ECG, propensity for atrial and ventricular arrhythmias,	unknown
*SCN5A*	Brugada syndrome	Elevation of the ST, ventricular fibrillation, syncope, arrhythmia	1:2000

For more details, see [11].

Sitosterolemia is a rare autosomal recessive disorder that is clinically similar to FH. Homozygous or compound heterozygous mutations in either *ABCG5* or *ABCG8* genes that lead to increased plant sterol (primarily sitosterol) absorption and plasma concentrations, xanthomas and accelerated atherosclerosis have been recognized as the molecular basis of the disease [12,13].

Marfan’s syndrome is an autosomal dominant disorder that has high penetrance with variable expression. It is caused by many different types of mutations in fibrillin 1 (*FBN1*) and manifests mostly as cardiovascular but also skeletal and ocular abnormalities. Affected individuals are usually very tall, and they have long limbs, long faces, long fingers and toes, hypomusculature, and chest, spine, hip, and foot deformities [14].

More than 100 monogenic CVDs have been identified, and at least 10 million people worldwide may have some of them [11] (for some examples see Table 1). Monogenic CVDs are characterized by early onset, usually severe symptoms, poor prognosis, and high mortality and disability rates. Therefore, monogenic CVDs cannot be underestimated, underdiagnosed, or undertreated. Fortunately, advances in gene testing technology, particularly next-generation sequencing, have triggered increased attention on the genetic diagnosis of “pre-symptomatic” subjects.

## 3. Association Studies

During the era of candidate gene studies, the genes and polymorphisms for analyses were selected according to our knowledge about the pathophysiology of the disease. This pioneering era was prone to many false positive (as well as false negative) results, and this was mainly due to the low numbers of examined subjects, which was often just a couple of dozen samples. Moreover, highly significant results were not confirmed in the following replication (often also small) studies.

However, some single genes/variants of unambiguous and high importance have been detected by this approach. Among them, polymorphisms within apolipoprotein E (*APOE*; OMIM acc. No. 107741) [15] as the determinant of plasma cholesterol levels, apolipoprotein A5 (*APOA5*; OMIM acc. No 606368) [16] as the determinant of plasma triglycerides, or melanocortin receptor type 4 as the determinant of body weight (*MC4R*; OMIM acc. No 155541) [17] shall be mentioned as successful and important examples of those days’ achievements.

## 4. Genome Wide Association Studies (GWAS)

After the era of candidate gene studies and chromosome mapping, GWAS significantly improved and extended our understanding of the genetic background of many noncommunicable diseases, including CVD and CVD risk factors [18]. The GWAS principle is “hypothesis free” [19]. This approach has led to the detection of many significant variants (followed by the identification of new genes) with completely unknown clinical importance.

The first GWAS dealing with CVD risk or CVD risk factors were published approximately 15 years ago [20,21,22], for review see [23].

GWAS have led to the detection of the most powerful genetic determinants of CVD, which are represented by SNPs (single nucleotide polymorphisms) within the human chromosome 9p21.3 region [24]. Variants were detected in a “gene-free” region (“gene desert”) later recognized as long noncoding regulatory RNA ANRIL (antisense noncoding RNA in the INK4 locus; located within the p15/CDKN2B-p16/CDKN2A-p14/ARF gene cluster) (OMIM acc. No. 613149) loci. The risk allele is associated with an increased risk of myocardial infarction (MI) by approximately 30–35%, and this effect is relatively homogenous in different populations and ethnic groups. Importantly, the detected variants are not associated with any of the traditional CVD risk factors (with the exception of diabetes), pointing at so far unknown mechanisms leading to atherosclerosis.

Interestingly, variants at these loci are associated with a wider spectrum of noncommunicable diseases. For example, associations have been shown for different types of cancer [25], glaucoma [26], or even autism [27]. These findings highlight the importance of these loci and the clustered SNPs as an exceptional genetic hotspot as a base of different diseases associated SNPs.

The second example of the “new” gene, which was detected due to GWAS, is sortilin (OMIM acc. No. 602458). GWAS signals within the *CELSR2/PSRC1/SORT1* gene cluster were among the strongest signals associated with the risk of CVD [21] and plasma levels of LDL cholesterol [28]. Additionally, in this case, the mechanisms by which SORT1 influences plasma cholesterol levels were (and remain in detail so far) unknown. *SORT1* encodes a multiligand sorting receptor that is expressed mainly in the liver and plays a role in intracellular trafficking. It binds to apolipoproteins E, B, and A5, which are important proteins acting in lipid metabolism (reviewed by [29]). SORT1 influences VLDL transport through the hepatic Golgi apparatus and simultaneously interacts with PCSK9 and influences LDLR degradation. Finally, it could serve as a low-capacity alternative LDL receptor (reviewed by [30]). However, although the effects of *SORT1* on plasma cholesterol levels and MI risk have been well proven and repeatedly confirmed, the results from animal models [31,32] are not uniform and sometimes show conflicting results. The roles of SORT1 seem to be very complex and controversial, and the exact molecular mechanism remains unclear. Thus, the essential answer has not been provided regarding the association of increased or decreased liver expression with an increased risk of hypercholesterolemia and higher CVD risk.

Despite the important role of *SORT1* in the determination of plasma cholesterol levels at the population level, a large study failed to detect any FH-associated mutation within this gene [33]. As the gene codes for the protein responsible for intracellular cholesterol transport, mutations within this gene may be severely deleterious and lead to lethal consequences in utero. This might explain why these mutations do not occur in the population.

At this point, we cannot omit *FTO*, which is probably the most interesting GWAS-detected gene and has gained multidisciplinary interest. GWAS demonstrated that *FTO* (“fat mass and obesity associated gene”; OMIM acc. No. 610966) was simultaneously associated with obesity [34,35] and an increased risk of type 2 diabetes mellitus (T2DM) development [36]. Variants in strong linkage disequilibrium clustering within the first intron of the gene are responsible for the increased risk. The associations with body mass index (BMI) and T2DM were quickly confirmed in later studies, and the association with BMI was described in all major ethnicities with the exception of black Africans, in whom the frequency of the risky allele and the proportion of BMI variation explained (reviewed by [37]) were much lower. The contribution of *FTO* variants to the risk of myocardial infarction was described shortly after the abovementioned first associations [38,39], followed by recognition of its role in renal failure [40], Alzheimer’s disease [41], diabetes complications [42], and even in the determination of total mortality [43].

Similar to ANRIL, the function of *FTO* is regulatory rather than structural or transport. A recent review [44] suggested that the variants exert their effects through another gene located in a cluster (*RPGRIP1-l/FTO/IRX3*) with *FTO*, namely, *IRX3* (Iroquois homeobox protein 3). *IRX3*, whose promoter binds to enhancers located within the 1st intron of *FTO* and whose expression is influenced by tagging *FTO* variants, has also been associated with body weight in animal models. *IRX3* is highly expressed in the pancreas, suggesting susceptibility to *FTO/IRX3* through the potential effect of insulin secretion.

Nevertheless, the roles of *FTO* as an important epigenetic modifier influencing nucleic acid methylation [45], telomere length determination [46,47], and serving as a transcriptional coactivator [48] have been described in the literature.

It can be concluded that the variants within the *FTO* 1st intron region are among the most important and interesting hits from the era of GWA studies.

It is important to note that the GWAS-detected variants within the known candidate genes from the “association studies” era are often different (and more powerful) than the original variants identified in association studies.

The selected examples of the most powerful and most interesting genes/SNPs playing a role in CVD determination or in the determination of CVD risk factors are listed in Table 2.

**Table 2:** Examples of genes which SNPs are associated with increased risk of cardiovascular disease (CVD) or with strong effect on CVD risk factors.

Gene	Effect on	Ref
*ANRIL*	Risk of myocardial infarction	[24]
*SORT1*	Plasma cholesterol levels	[21,28]
*APOA5*	Plasma triglyceride levels	[16,49]
*FTO*	BMI values, risk of T2DM and myocardial infarction	[34,35,36,38,39]
*TCF7L2*	Risk of T2DM	[50]
*APOE*	Plasma cholesterol levels	[15]
*MC4R*	BMI values	[51]
*CHRNA5-A3-B4*	Smoking addiction	[52]
*UMOD*	Hypertension	[53]

## 5. Gene Score

### 5.1. Polygenic Predisposition

As mentioned above, single SNPs have a relatively minor effect on disease risk (usually with OR between 1.05–1.50 per allele) or just a slight effect on biochemical/anthropometrical parameters. For example, they are associated with increasing plasma total cholesterol by approximately 0.10–0.35 mmol/L; body weight by max. approximately 500–700 g per risky allele or blood pressure by 2–5 mmHg.

**Figure 2:**
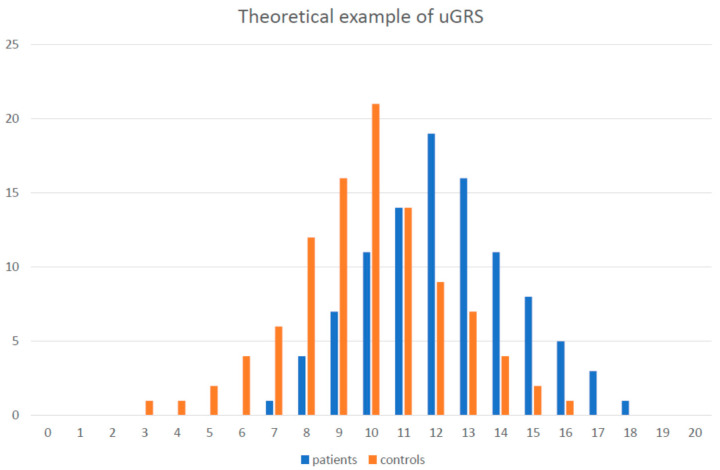
Theoretical example of gene score construction. Theoretical example of unweighted gene score (based on sum of the risky alleles) in group of patients and controls. With 10 genes (polymorphisms) the values could be between 0 and 20. In fact, the lowest and the highest values are almost never presented. There is significant shift in the distribution curve in patient groups to the higher numbers of risky alleles. Still, there is also significant overlap between the patients and controls in gene score values.

Although it is clear that noncommunicable diseases have polygenic backgrounds [54], the number of studies focused on polygenic determination of CVD is limited. Nonetheless, the published results show that polygenic scores represent the right approach for the personalized management of diseases (Figure 2).

An analysis of “only” one SNP is undoubtedly important to describe its potential in the determination of human phenotype/disease. However, such analyses, albeit of importance and interest, do not represent unquestionable proof and are not sufficient for identifying the risk factors leading to disease development. To overcome this problem, simultaneous analysis of more SNPs and the calculation of so-called gene risk scores or genetic risk scores (GRSs) or polygenic risk scores (PRSs) seem to be a reasonable approach [55,56]. A variable number of polymorphisms are being used for gene score construction, from a few to thousands.

### 5.2. Unweighted and Weighted Genetic Risk Score

To calculate the GRS, alias PGS, two approaches are being used. The simple unweighted GRS (uGRS) summarizes the number of risk alleles, regardless of the effect size of each allele, and the weighted GRS (wGRS) takes into account the real effect of the SNP on the risk/biochemical parameter. Thus, for example, ANRIL variants will have higher power than risky alleles within other genes.

Both approaches have some advantages and pitfalls. The disadvantage of the wGRS is that the exact effect of the SNP on the parameter of interest within the population must be known for further calculations. This could be problematic because not all effects are necessarily generally applicable, even within populations of identical or similar ethnicities, as others and we have shown for the *MLXIPL* gene and plasma triglyceride values [57,58,59]. The wGRS could also be influenced by age, sex, and a wide list of environmental factors.

In contrast, for the simple uGRS, the exact effect of each genotype/allele does not have to be known, and information about the risky status is sufficient, although even this could be confusing, especially between the different ethnicities. For example, Japanese researchers [60] included *AB0* gene variants in the GRS used for plasma lipid level estimation. Variants within this gene were not reported as significant in GWAS performed on Caucasians. An identical study that used different variants within the genes for *SORT1*, *LDLR*, and *HMGCR* showed that the effect sizes for the genes were different in comparison to the Global Lipids Genetics Consortium score (http://lipidgenetics.org accessed on 5 March 2021).

Given that the calculation of the GRS shall be used for disease risk estimation in young, asymptomatic subjects (to allow the detection of subjects at risk as soon as possible to initiate intensive and focused lifestyle interventions, potentially followed by pharmacological treatments), the use of the uGRS seems to be a sufficient tool.

### 5.3. GRS Examples

Initial studies focused on simultaneous analyses of several polymorphisms in association with plasma cholesterol levels were published (without attracting much attention) more than a quarter of century ago [61,62] and included *APOE*, *APOB*, and *LDLR* genes.

The golden age of the gene scoring began after the description of the human genome and after wider availability of high-throughput genotyping technologies. Briefly, there is a wide list linking the GRS and obesity prediction [63], plasma TG values [64,65], or hypertension [66].

Talmud et al. [67] used a preselected list of 65 SNPs to estimate the risk of T2DM development. Although the individual odds varied between 0.93 and 1.32, if the subjects from the top vs. bottom quintiles of the gene score were compared, the 10-year risk of T2DM development was almost 3 times higher for the top-quintile subjects.

Very detailed and extensive analyses on the identical phenotype were performed in the Estonian Biobank cohort [68]. Two different types of gene scores were used: Weighted and double weighted (taking into account the P values from all available studies for the SNPs of interest). Furthermore, different numbers of SNPs (from 65 to 2100) were used in different models, as well as different types of adjustments. Interestingly, the best prediction for T2DM manifestation was reached with the GRS that included 1000 SNPs and in the model without adjustment for BMI.

Using the 53 SNPs, Morris et al. [69] did not significantly improve CVD risk predictions based on traditional risk factors. Some utility has been demonstrated for intermediate-risk groups only, which was not surprising because a significant number of the selected SNPs used for the risk estimation were associated with traditional risk factors, mainly dyslipidaemia. Moreover, the effect of some individual SNPs used for GRS calculation was not confirmed in this study, thus highlighting the risk of inaccuracies, if external values are used for the calculation.

GRS is also used for the detection of so-called “polygenic FH” (see above), although FH was originally recognized as a monogenic disease. Currently, approximately 20% of clinically diagnosed FH cases are suggested to have the polygenic form of FH [6,70]. FH patients without monogenic causes have been shown to have a high GRS resulting from the contribution of several (usually 6–12) common LDL-C-raising SNPs.

A study published by Khera et al. [71] can serve as an extreme example of the use of the GRS in CVD prediction. The authors used more than 6 million SNPs for the risk estimation, and the high GRS was associated with a 3.7-fold increased likelihood of myocardial infarction. The cost–benefit of such analysis is thus questionable because the strongest CVD-associated SNPs within the *ANRIL* loci (see above) have ORs of approximately 1.7 in the case of homozygosity.

Thus, including such extremely high numbers of SNPs will not add as much value as expected; moreover, the optimum has not been identified.

## 6. Nutrigenetics

In the vast majority of noncommunicable diseases, neither the genes nor the environment or lifestyle individually are sufficient to fully explain the risk of the disease. Thus, we need to improve our understanding of the interactions between the genes and environment, especially between genes and diet.

From environmental and lifestyle factors, an unhealthy diet (beyond smoking) is one of the leading causes of mortality and morbidity worldwide. In fact, the Global Burden of Disease project estimated that in 2015, nutritional risk factors were responsible for more deaths and disease worldwide (calculated as % of disability adjusted life years—DALY) than any other risk [72]. An earlier analysis from the WHO found that an unhealthy diet together with physical inactivity contributed to 57% of cardiovascular deaths globally (//apps.who.int/iris/handle/10665/44203, accessed 5 March 2021). Genetic determinants of eating habits have been the focus of considerable scientific interest for decades.

An important area of research in this topic is to identify genetic targets for the purpose of personalized medicine and personalized nutrition. The interaction between dietary habits and genes is extremely complex and can go both ways: Specific genetic characteristics can influence the effect of the diet on biochemical or anthropometrical characteristics (nutrigenetics); however, what we eat may also have an impact on gene expression (nutrigenomics).

Within nutrigenetics, there are several illustrative examples showing how SNPs could influence the effect of consumed nutrients on plasma lipids or body weight.

Cholesterol 7 alpha-hydroxylase (CYP7A1) is the rate-limiting enzyme in the bile acid synthesis cascade, the only metabolic pathway for cholesterol elimination from the body [73]. There are several functional polymorphisms within the *CYP7A1* promotor region. In a primary study, rs3808607 significantly influenced cholesterol decrease in a subset of males in the Czech population based post-MONICA study after marked changes in dietary habits (caused by socioeconomic changes between 1988 and 1996) and suggested the highest sensitivity of CC homozygotes [74]. The results were later confirmed in interventional studies based on dietary cholesterol intake in male volunteers [75] and in randomized trial focused on plant sterol consumption [76] or interventions based on cafesterol consumption [77].

The abovementioned *FTO* gene interacts with many dietary factors. Subjects with low protein intake and the “BMI-increasing” allele of *FTO* have the highest BMI values, and this interaction is particularly strong in distinct Asian populations [78]. The modification of the effect of *FTO* on BMI values through protein intake has also been found in children and adolescents [79]. Finally, higher birth weight also attenuated an association between the *FTO* genotypes and BMI values in adulthood [80].

Homocysteine, which is believed to be a risk factor for MI and stroke, is associated with the dietary intake of folate. It has been shown that the effect of methylene tetrahydrofolate reductase (*MTHFR*) polymorphism on homocysteine concentration is larger in regions with low folate intake than in regions where folate fortification is common [81].

The abovementioned risk associated with variants within the *ANRIL*, long noncoding regulatory RNA, although not associated with traditional risk factors, could be modified by dietary habits. It has been described [82] that increased intake of sugar-sweetened beverages interacts with *ANRIL* variants. An increased risk of nonfatal MI was observed only in subjects consuming more than 2 servings daily. Interestingly, this interaction was not observed in consumers of fruit juice only, despite identical energy intake per servings.

The list of potentially interesting nutrigenetic associations is long; however, a number of results remain unconfirmed in subsequent independent studies. The list of reviews and opinion papers on the topic is probably as long as the list of original results, but some of them are useful to read [83,84,85].

The significant lack of knowledge in the field of nutrigenetics and lack of a description of real and valid gene-environment interactions are much more complicated than originally believed.

In one of our previous studies, we focused on the determination of binge drinking and alcohol consumption within the large HAPIEE study, which involved almost 30,000 subjects from four countries [86]. For the differentiation between the subgroups of binge drinkers and controls, the two genes of interest (*FTO* and *ADH1B*), sex, smoking status, and drinking pattern were gradually included in the model and examined. Discrimination into these subgroups has resulted in subgroups with approximately 200–500 subjects. Thus, even the use of very large, precisely defined, and extensively and homogenously phenotyped subjects cannot guarantee unquestionable results and final answers, and such studies need to be replicated in independent cohorts.

## 7. Pharmacogenetic of CVD Treatment

The metabolism, effects, and side effects of all drugs are being determined and/or modified by genetic background. Pharmacogenetics will help us understand the complex relationships and determine individualized/personalized approaches to pharmacotherapy. There is significant interpatient variability in drug response, much of which has a genetic basis. Specifically, genotypes can influence drug metabolism, drug transport, and a person’s sensitivity to a drug [87].

In pharmacogenetics, the interactions between genetic variants and drug efficacy and/or susceptibility to undesirable side effects (USEs) are analyzed. This field has the potential to improve health outcomes by identifying individuals who have a greater benefit from treatment or who are at greater risk of harm caused by medication intolerance [88].

Statins are inhibitors of hydroxymethylglutaryl CoA-reductase (key enzyme of cholesterol biosynthesis), and they are among the most frequently used drugs. Despite the relatively low USE prevalence, the numbers of affected subjects are huge because the number of patients taking statins is enormous.

There are several genes with the potential to be helpful in the detection of subjects prone to statin USE (mostly muscle symptoms) [89,90,91].

The most powerful transporter seems to be the *SLCO1B1* drug transporter. The gene encodes an organic anion-transporting polypeptide OATP1B1, which regulates the hepatic uptake of statins. A common variant (rs4363657) within this gene is associated with a significantly increased risk of myopathy, and carriers of two risk alleles have a 16-fold increased risk [92]. Later studies suggested [93,94,95] that this risk is limited to subjects on high statin doses (at least 40 mg daily), especially to patients treated with simvastatin.

For the treatment of hypertension, an important drug class is beta-blockers. It is recommended to examine the variants within *CYP2D6* for the optimal dosing and avoiding the USE of this drug class [96].

## 8. Epigenetics

The term epigenetic has been several times redefined and recently, the most accepted definition is that epigenetic information represents the type of genetic information, which is not directly included in the DNA sequence. There are several mutual characteristics of epigenetic factors. They are reversible and not stable across the entire life span of individuals, and they can be influenced by the subjects’ lifestyle (e.g., by physical activity, smoking status, dietary habits). At least some of them (DNA methylations) are clearly heritable similarly to the DNA sequence. Epigenetic changes have primarily regulatory effects. There are two major types of epigenetic information [97] potentially associated with CVD that are commonly analyzed; (i) regulatory noncoding RNA, mostly represented by microRNA (miRNA) and (ii) DNA methylation.

Despite the modification of histones (mostly acetylation, methylation. and phosphorylation), which can potentially affect DNA replication, chromosome condensation or alternative gene splicing could have important consequences in connection with CVD [98], there is a significant lack of concise and well-performed studies.

### 8.1. Regulatory Non-Coding miRNA

MicroRNAs (miRNAs) are endogenous, short noncoding single-stranded RNA molecules, ~22 nucleotides in length, which act as transcriptional regulators and are involved in posttranscriptional processes.

MiRNAs are derived from transcripts that form distinctive hairpin structures. Processing of the hairpin into the mature miRNA allows the formation of an RNA-induced silencing complex (RISC) [99]. The miRNAs then pair with targeting mRNAs by binding to the different gene regions: 3′-untranslated region (3′UTR), 5′UTR, promoter or coding sequences. miRNA binding can both repress or activate translation. The crucial binding location for translational regulation resides in the mature miRNA sequence, called the seed region [100,101].

Deregulation of miRNA expression plays a crucial role in the pathogenesis of numerous diseases, including cardiovascular diseases. Changes in miRNA expression and/or function have been associated with various cardiovascular complications, such as myocardial infarction, cardiac hypertrophy, cardiomyopathy, or arrhythmias [102,103]. MiRNAs are expressed in cardiomyocytes, fibroblasts, endothelial cells, and vascular smooth muscle cells and control virtually all aspects of cardiovascular biology, including cardiac remodeling and fibrosis, apoptosis, inflammation, proliferation, angiogenesis, and metabolism [104].

MicroRNAs play a critical role in myocardial infarction by regulating apoptotic, necrotic, and autophagic cell death. Many miRNAs are differentially regulated in heart tissue in response to myocardial infarction depending on the type of myocardial injury [105].

MiRNAs are involved in every stage of the biological process of atherosclerosis, that is, endothelial dysfunction, cellular adhesion, plaque development, and plaque rupture (Table 3). Moreover, endothelial cells (ECs), macrophages, and smooth muscle cells (SMCs), which participate in pathways of plaque and thrombus formation, may potentially release miRNAs in systemic circulation [106].

**Table 3:** List of selected regulatory miRNA involved in CVD development.

miRNA	Function
miR-15 family, miR-34 family, miR-499, miR-320, miR-24, miR-1, miR-16, miR-21, miR-92a, miR-375, miR-103/107, miR-133a/b, miR-214	Differently regulated in heart tissue in response to myocardial infarction
miR-34a, miR-217, miR-146a	Endothelial cell senescence
miR-126, miR-31, miR-17-3p	Vascular inflammation
miR-21, miR-221, miR-222, miR-143/145 cluster, miR-1, miR-10a	SMC (smooth muscle cell) differentiation, survival, proliferation, and dedifferentiation
miR-155, miR-125a-5p	Monocytes/macrophages lipid uptake and inflammatory responses
miR-146a, miR-128, miR-365, miR-503	Effect on migration of macrophages
miR-33, miR-302a, miR-122, miR-370, miR-335, miR-378, miR-27, miR-125a-5p, miR-33a/b, miR-144, miR-223, miR-148a, miR-128-1	Cholesterol homeostasis and fatty acid oxidation

For more details, see [100,101,102,103,104,105,106,107,108,109,110,111,112,113,114].

Several miRNAs have been associated with regulatory mechanisms involved in EC senescence [107,108] and regulate vascular inflammation [109,110]. In addition to proinflammatory cytokines, changes in miRNA expression levels due to blood flow have the potential to affect networks of genes regulating endothelial and vascular smooth muscle cell function, inflammation, and atherosclerosis [111].

In response to endothelial dysfunction and inflammatory cell infiltration, SMCs migrate from the media to the intima and proliferate to form neointimal lesions. The switch from a contractile to a synthetic proliferative phenotype in SMCs is controlled by miRNAs, some of which are essential for the acquisition of the contractile phenotype, SMC differentiation, and the structural integrity of the aorta [112]. MiR-133 was reported to prevent the phenotypic switching of SMCs, and miR-21, miR-221, and miR-222 promote SMC survival, proliferation, and dedifferentiation [113].

Lipid uptake and inflammatory responses in monocytes/macrophages are regulated by miRNAs, such as miR-155 and miR-125a-5p [114,115]. As a result, neointimal accumulation of foam cells and fatty streaks can be reduced, which is a main determinant of plaque development and instability.

MiRNAs regulate signaling and lipid homeostasis pathways that alter the balance of atherosclerotic plaque progression and regression. MiRNAs have been identified to be potent posttranscriptional regulators of genes involved in the regulation of cholesterol homeostasis and fatty acid oxidation, fatty acid metabolism, and lipogenesis [116], and have been shown to play a key role in governing HDL metabolism [117].

Circulating miRNA released from cells can be detected in virtually all human body fluids [118]. Unlike intracellular miRNAs, circulating miRNAs show remarkable stability and resistance to degradation by endogenous RNases. MiRNAs can be released into the blood circulation by various mechanisms, including active secretion, apoptosis, or necrosis. It has been proposed that circulating miRNAs reside in microvesicles, which may provide protection from RNase activity and account for the shedding of miRNAs into the circulation [119]. Cell-secreted miRNAs play an important role in cell-to-cell communication. The stability of circulating miRNAs has stimulated interest in their use as biomarkers for the diagnosis and prognosis of various diseases, including CVD.

### 8.2. DNA Methylation

DNA methylation seems to be the most important and most extensively studied DNA epigenetic modification in association with CVD [120]. DNA methylation affects cytosine within CpG sites across the entire genome. Of all the epigenetic markers, DNA methylation is the most stable and occurs approximately within every second gene, and 70% of all CpGs within the human genome are methylated [121]. Generally, the global DNA methylation pattern can be analyzed: Methylation within long-interspersed nuclear elements (LINE-1) or within ALU sequences. Finally, detailed differences near the candidate genes within their regulatory elements could be analyzed using comparative bisulfite sequencing. Epigenetic changes could lead to an increase in both the transcription machinery and gene silencing. The importance of appropriate nutrition is underlined here—the sufficient intake of methyl-group donors, such as folate and other B vitamins, is necessary for optimal epigenetic regulation [122].

Agha and colleagues [123] performed a large epigenome-wide profiling study and detected that out of the almost 500,000 CpGs, the methylation levels at more than 50 CpG sites were significantly associated with incident CVD or myocardial infarction. These CpG sites mainly influenced genes involved in calcium homeostasis and calcium-dependent regulation, cardiac remodeling, and leukocyte transendothelial migration.

The importance of prenatal nutrition for the development of CVD in adulthood was suggested in a study in which six loci sensitive to prenatal nutrition were examined [124]. Increased methylation at GNASAS (alias NESPAS, with a highly imprinted expression pattern involved in the regulation of fetal nutrient demand) was associated with an increased risk of MI in females.

The results from the German KORA study [125] pointed to the importance of DNA methylation for the determination of plasma lipids. Interestingly, it seems that many more CpG sites are involved in the determination of plasma triglycerides and total cholesterol than in the determination of LDL and HDL cholesterol levels.

## 9. Telomeres

Telomeres are nucleoprotein complexes located at the ends of the linear chromosomes, and they protect chromosomes from DNA degradation and fusion. In humans, telomeres consist of thousands of TTAGGG hexanucleotides and a protein complex called shelterin. Telomere length is used as a marker of biological/cellular age [126], as telomeres become shorter with each cell division. It is worth mentioning that most studies analyze the relative telomere length, not the absolute telomere length.

Shorter telomere length was associated with an increased risk of cardiovascular mortality in several large longitudinal studies [127,128]. Additionally, some CVD risk factors were associated with telomere length, such as obesity, plasma lipids, or hypertension. These associations could be influenced by the age of examined subjects and generally showed strong heterogeneity effects [129,130,131].

It is important to remember that in contrast to SNPs or mutations, the pattern of epigenetic changes, unfortunately, will not be identical in different tissues [132,133,134]. Thus, for example, the relevance of information obtained from DNA isolated from peripheral blood (which is the most common source of DNA) for cardiac cell function is not absolutely justified.

## 10. Geographical and Ethnical Differences

Different populations and ethnicities have significant differences in genetic backgrounds, and these differences may influence disease development.

Rare mutations are geographically specific, which is not surprising because the prevalence differences are based often on the “founder effect”. However, important and extraordinary differences are also observed for allelic frequencies of many common and important variants.

At this point, apolipoprotein L1 (*APOL1*, OMIM acc No. 603743) and its variants should be mentioned. The *APOL1* gene encodes an ion channel, and there are two known functional variants (rs73885319 and rs71785313). These variants are associated with an approximately twofold-fold increased risk of CVD (and even with a 10-fold increased risk of renal failure) [135]. The spread of these variants is a result of their primary function: Protection against *Trypanosoma brucei* infection (cause of sleeping disease) [136]; thus, as a result of selection pressure, they occur in black Africans only [49].

Additionally, variants within the abovementioned *APOA5* gene show strong interethnic differences (for review see [137]). For example, one of the variants (rs3135506) commonly present in almost 30% of Hispanics and in approximately 14% of Caucasians and Africans is almost completely missing in Asians (less than 1%). In contrast, the minor allele of the *APOA5* variant rs2075291 is present in Asians only.

Significant differences could also be observed within the large ethnic groups, despite hundreds of years of inhabiting identical geographic regions. The largest Middle European minority are Roma/Gypsy subjects, exhibiting identical ethnicity as the majority—Caucasians. Between Roma Caucasians and non-Roma Caucasians, significant differences were described (not only) in genetic predisposition to T2DM, albeit in both directions depending on the set of SNPs selected [138,139].

Another example of interethnic difference originating from nutrition-based selection pressure is the rs1229984 variant within the alcohol dehydrogenase (*ADH1B*, OMIM ac. No. 103720) gene. The enzyme encoded by *ADH1B* is the key metabolizer of ethanol, and two isoforms (with His or Arg at amino acid position 47) exhibit highly significant differences in ethanol catabolism. Slow metabolizers (Arg carriers) consume approximately 30% more ethanol than rapid Arg metabolizers [140]. This paradox is caused by the fact that rapid metabolizers accumulated the first ethanol degradation product, acetaldehyde, which causes unpleasant reactions to alcohol consumption. There are extreme allelic differences between Europeans (~5% of the His allele) and Asians (~80% of the His allele) [141], and the differences are considered the result of selection pressure in the past. Increased aldehyde concentrations associated with allele His help to degrade toxins, products of rice decay. Due to the medieval era, most European inhabitants consumed beer and wine because water was highly sludge-contaminated; however, in Asia, this problem was solved by boiling water, and tea was preferably consumed.

The abovementioned examples clearly point to the fact that the use of general GWAS results for calculation could be misleading, especially when different ethnicities and males or females or subjects from different geographical regions with different lifestyle habits are examined.

## 11. Heritability of CVD risk Modifiers: Less Understood Piece of the Puzzle

The list of the CVD risk factors or factors associated with CVD seems to be endless [142], and at least partial genetic determination can be assumed for all risk factors as well as risk modifiers, which was suggested by the latest versions of ESC/EAS guidelines [143]. However, studies evaluating the genetic component of complex risk modifiers and factors known to be associated with increased CVD risk (e.g., socioeconomic factors, climate, psychosocial factors, etc.) are infrequent and difficult to conduct and evaluate and interpret [144].

## 12. Conclusions and Future Direction

“One size will not fit all” is the basis of personalized medicine, which has been promulgated over the last decade at conferences and meetings and fills the pages of scientific journals. Unfortunately, large gaps remain in clinical practice, and the motivation for bringing this approach closer to patients may also be lacking [145].

Although nutrigenetic and nutrigenomic research has made some notable discoveries over the last years, such as identifying genetic determinants of obesity, lipid metabolism, or type 2 diabetes [146,147,148], the application of these findings in clinical or public health practice is also infrequent. Nonetheless, despite the methodological challenges, this line of research has the potential to offer practitioners the opportunity to provide dietary advice that is specifically tailored to the needs of the individuals. Unfortunately, such an approach is more often detectable at the private level and rarely supported by health insurance companies.

Determining the genetic determinants of our response to diet or treatment could be important for a number of reasons for both clinical and public health research and practice.

We can measure the genes, improve the accuracy of dietary assessments, and reduce the impact of measurement bias and residual confounders, which are often major limitations in traditional nutritional epidemiological analyses that could help to tailor more detailed recommendations. Although enormous amount of genetic information is available, more extensive assessment of such information relative to other traditional risk factors have not been performed; thus, genetic information has not been adequately used for CVD risk estimation.

Explosive progress achieved in understanding the rapidly evolving science underlying CVD genomics has resulted in fee-for-service testing, making genetic information widely available. Proper interpretation of these results is even more important than ever before. The power of genetic analysis lies most prominently in screening family members at risk for developing disease and excluding unaffected relatives, which is information not achievable otherwise. Genetic testing also allows expansion of the broad underlying inherited risk conditions spectrum and precise diagnosis of pathologies with different natural history and treatment options. Interfacing a heterogeneous disease such as CVD with the vast genetic variability of the human genome, and high frequency of novel mutations, has created unforeseen difficulties in translating complex science (and language) into the clinical arena. Indeed, proband diagnostic testing is often expressed on a probabilistic scale, which is frequently incompatible with clinical decision making. Major challenges rest with making reliable distinctions between pathogenic mutations and benign variants, and those judged to be of uncertain significance. Genotyping in CVD risk prediction can be a powerful tool for family screening and diagnosis. However, wider adoption and future success of genetic testing in the practicing cardiovascular community depends on a standardized approach to DNA variability interpretation, and bridging the communication gap between basic scientists and clinicians.

## Data Availability

Not applicable.
